# Quantum Control and Quantum Information Technology

**DOI:** 10.1155/2013/525631

**Published:** 2013-09-19

**Authors:** Daoyi Dong, Chunlin Chen, Min Jiang, Lin-Cheng Wang

**Affiliations:** ^1^School of Engineering and Information Technology, University of New South Wales at the Australian Defence Force Academy, Canberra, ACT 2600, Australia; ^2^Department of Control and System Engineering, Nanjing University, Nanjing 210093, China; ^3^School of Electronics & Information Engineering, Soochow University, Suzhou 215006, China; ^4^School of Physics and Optoelectronic Technology, Dalian University of Technology, Dalian 116024, China

Quantum information technology has been recognized as one of the most promising future technologies (e.g., see [1]). The development of quantum control theory is a key task for practical quantum information technology (e.g., see [2–4]. Recent advances in quantum information technologies and robust quantum information processing require the dynamics of realistic quantum systems to be characterized and controlled with unprecedented accuracy and efficiency. The interactive promotion between the two fields is a rapidly developing research area. The purpose of this special issue is to provide an account of the state of the art in this fast moving and cross-disciplinary field. 

18 papers have been received for this special issue and 9 among them have been accepted for publication. The paper by C. Chen et al. presents a survey on the recent development of closed-loop control strategies in quantum control including closed-loop learning control and quantum feedback control as well as an introduction to quantum robust control. The paper by S. Cong and F. Meng presents a survey on Lyapunov control of quantum systems which is a “feedback design” and “open-loop control” strategy. The paper by S. N. Khonina et al. develops an algorithm for solving the steady-state Schödinger equation for a class of potentials. The paper by S. Zhao et al. proposes a hybrid impulsive control method combined with Lyapunov control to achieve a more accurate convergence for a class of nonideal quantum systems. The paper by V. Tiporlini and K. Alameh investigates the principles of operation and the optimal design of an optically pumped quantum magnetometer. The paper by W. Yang and J. Sun investigates the Lyapunov control of finite-dimensional quantum systems with impulsive control fields. The paper by M. Li et al. proposes a feedback control scheme through Rabi oscillation stabilization to preserve the coherence in cavity QED. The paper by Y. Xing and J. Wu presents a quantum control method to control the Shannon entropy of quantum systems. The paper by M. Li proposes a numerical simulation algorithm for stochastic process of direct photodetection of a driven two-level system.
